# Impact of nutritional status and pulmonary function on short- and long-term overall survival in hemodialysis patients

**DOI:** 10.1371/journal.pone.0317510

**Published:** 2025-01-27

**Authors:** Özge Aydın Güçlü, Hasim Atakan Erol, Nilüfer Aylin Acet Öztürk, Asli Gorek Dilektasli, Funda Coskun, Abdulmecid Yıldız, Mehmet Karadag

**Affiliations:** 1 Department of Pulmonary Diseases, Uludag University Faculty of Medicine, Bursa, Turkey; 2 Department of Haematology, Kocaeli University Faculty of Medicine, Kocaeli, Turkey; 3 Department of Nephrology, Uludag University Faculty of Medicine, Bursa, Turkey; Shuguang Hospital, CHINA

## Abstract

**Background:**

End-stage renal disease (ESRD) patients frequently experience protein-energy wasting (PEW), which increases their morbidity and mortality rates.

**Objective:**

This study explores the effects of nutritional status and pulmonary function on the short- and long-term mortality of ESRD patients undergoing hemodialysis.

**Materials and methods:**

67 consecutive ESRD patients on maintenance hemodialysis were included in the study. The primary outcomes were all-cause one-year and five-year mortality. Data on demographic characteristics, comorbidities, and laboratory findings were collected. Pulmonary function tests were conducted along with body composition measurements using bioelectrical impedance analysis (BIA). Malnutrition was assessed using the Prognostic Nutritional Index (PNI).

**Results:**

The median age of the patients was 60.9 ±  12.4 years, with 58.3% being male. Pulmonary function parameters (FEV1 and FVC) were significantly associated with short-term mortality. The PNI was a significant predictor of both short-term and long-term mortality. A PNI score ≤  39.01 was associated with increased short-term mortality (HR: 0.65, 95% CI: 0.48–0.88, p =  0.006), while a score ≤  40 was linked to increased long-term mortality (HR: 0.80, 95% CI: 0.67–0.95, p =  0.015). Additionally, older age (HR: 1.06, 95% CI: 1.01–1.12, p =  0.021) and higher glomerular filtration rate (GFR) (HR: 1.23, 95% CI: 1.02–1.42, p =  0.024) were related to increased long-term mortality risk.

**Conclusion:**

The study demonstrates that PNI, age, and pulmonary function are critical factors influencing the survival of hemodialysis patients. These findings underscore the importance of comprehensive nutritional and pulmonary assessment to improve clinical outcomes in this population.

## Introduction

A global public health concern, chronic kidney disease (CKD) is characterized by an increasing incidence and prevalence, a poor prognosis, and significant medical costs [1]. Protein-energy wasting (PEW), defined as the combined loss of body protein and energy storage, is a prevalent symptom in people with CKD and end-stage renal disease (ESRD) [2]. Poor clinical outcomes, such as increased morbidity and mortality, are associated with PEW [3]. Malnutrition in hemodialysis patients is multifactorial and can be defined using different tools [4]. The Prognostic Nutritional Index (PNI), generated from serum albumin levels and lymphocyte count, has been used to evaluate patients’ nutritional and immunological conditions [5]. The serum albumin concentration is a significant indicator of nutritional status, with hypoalbuminemia linked to poor outcomes in ESRD patients [6]. Additionally, lymphocytes, crucial for cell-mediated immunity, are associated with prevalent atherosclerotic cardiovascular disease in ESRD [7].

Lung impairment is another major problem among ESRD patients. This group of individuals has been diagnosed with a range of pulmonary abnormalities, such as pulmonary edema, pleural effusion, acute respiratory distress syndrome, pulmonary hypertension, pulmonary calcification and fibrosis, hemosiderosis, pleural fibrosis, and obstructive sleep disorder [8]. Lang et al. studied spirometric changes thoroughly before and after hemodialysis (HD) and found no significant difference in pre-HD and post-HD vital capacity [9]. Evaluating lung dysfunction’s influence on ESRD patients’ survival is essential for improving clinical management and patient outcomes. The impact of malnutrition and pulmonary function test values on short and long-term mortality in end-stage renal illness has not been investigated.

Our research aimed to assess the impact of lung function and the PNI on the short- and long-term mortality of ESRD patients.

## Method

### Study design and population

This retrospective cohort study included 67 consecutive patients with ESRD treated with maintenance HD at Boyabat 75th Year State Hospital between 01/10/2019 and 30/10/2019. The first data for research purposes was accessed on 01/11/2023.

**Fig 1:**
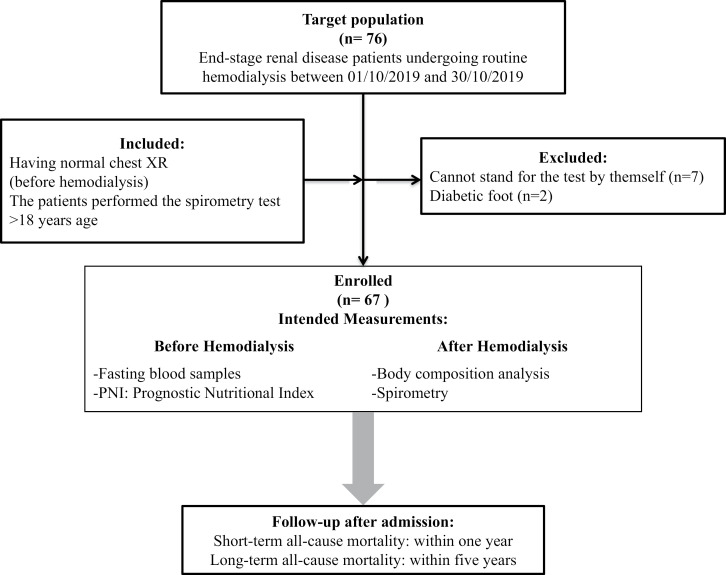
Study flow chart.

The inclusion criteria were having a normal chest X-ray (before hemodialysis), patients who performed the spirometry test, and an age of at least 18 years. Patients who cannot stand for the test by themselves, have an acute respiratory infection, decompensated congestive heart failure, cardiac valve disease, hypertension ( > 180/100 mm Hg), arrhythmia, amputated lower limb, or diabetic foot were excluded (Fig 1).

Demographic data (sociodemographic characteristics, smoking history, comorbidities, dialysis duration as a month, and etiology of renal failure) were recorded. The study was conducted by the Declaration of Helsinki and received approval from the Sinop University Local Ethics Committee (approval number: 2020/11). All data were fully anonymized before access, and the ethics committee waived the requirement for written informed consent since it was designed retrospectively.

### Variables

The primary outcomes were all‐cause short‐term (within six months) and long-term (within five years) mortality. Patients were monitored for death or survival until November 10, 2023. Electronic medical records at the clinic provided information about the date of death and the vital state of the patients.

### Hemodialysis

The patients underwent an average of three HD sessions per week, each lasting three to four hours, using an arteriovenous fistula. These HD sessions utilized a Hospital Integra monitor and a dialyzer with a 2 m² polyarylethersulfone membrane (Fresenius 4008S dialysate).

### Laboratory evaluation

Fasting blood samples were obtained before HD. For the laboratory evaluations, serum phosphorus, albumin, transferrin, pro-b-type natriuretic peptide (proBNP), hemogram, and lipid profile were recorded.

### Body composition analyses

We employed bioelectrical impedance analysis (BIA), an alternative to conventional anthropometric approaches to dietary monitoring. In HD patients, body composition tests were performed 30 minutes following a euvolemic dialysis period. Body composition was evaluated using the Tanita BC-420MA Body Composition Analyzer (Tanita, Tokyo, Japan). The subjects stood straight on the analyzer footpads in their bare feet for the BIA measurements. The lower body was exposed to an alternating current at 50 kHz and 200 mA, while the impedance between the two feet was recorded. The body mass index (BMI), visceral fat ratios, total body water, fat-free mass, muscle mass, fat mass, and fat ratio were computed and reported for each patient. The fat-free mass index (FFMI) was calculated using the equation: FFMI =  fat-free mass [kg]/(height [m]²), expressed in kilograms per square meter (kg/m²).

### Spirometry

The quality of spirometry tests was assessed based on the guidelines provided by the American Thoracic Society (ATS) and the European Respiratory Society (ERS). These guidelines outline criteria for determining the acceptability and reproducibility of spirometry maneuvers. For a test to be considered acceptable, it must include a proper start, continuation, and conclusion of the maneuver.

Acceptable tests should not exhibit artifacts such as coughing during the first second of exhalation, glottis closure, spirometer leakage, or obstructions in the mouthpiece. The exhalation phase must last at least six seconds, demonstrate a plateau on the volume-time curve, and have an appropriate start (extrapolated volume less than 5% of the forced vital capacity [FVC]).

At least three acceptable maneuvers were performed to ensure reliability and confirm repeatability. This required that the difference between the highest two FVC values and the highest two forced expiratory volume in one second (FEV1) values did not exceed 150 ml [10]. The forced vital capacity (FVC), forced expiratory volume (FEV1), and forced expiratory volume ratio (% FEV) parameters were evaluated.

### Malnutrition evaluation

The Prognostic Nutritional Index (PNI) was utilized to conduct an objective nutritional assessment. The formula used to calculate the PNI was (10 ×  serum albumin (g/dL) +  0.005 ×  total lymphocyte count). A score of more than 38 is considered normal, while scores between 35 and 38 and less than 35, respectively, indicate moderate and severe malnutrition [5].

### Statistical analysis

The data was analyzed using IBM SPSS Statistics for Windows, Version 28.0. and p  <  0.05 was considered statistically significant. The Kolmogorov-Smirnov test was utilized to evaluate if the variables were normally distributed. The continuous data were presented as mean, standard deviation, median, and interquartile range (IQR) values, while the categorical variables were expressed as n (%). For between-group comparisons, parametric independent sample t-tests or non-parametric Mann-Whitney U tests were used depending on the findings of the normality test. Pearson’s Chi-square test was performed to compare categorical variables. The multivariate Cox regression model was constructed with the variables that met the p < 0.25 threshold in univariate analysis. The variables were selected using the backward stepwise LR approach, and the analysis’s findings were presented. A summary was provided for the hazard ratios (HRs) and 95% confidence intervals (95% CIs). MedCalc Statistical Software (version 20.026, 2022; Ostend, Belgium; https://www.medcalc.org) was used to analyze receiver operating characteristics (ROC). The effectiveness of the PNI total score in predicting short- and long-term mortality was assessed using an ROC curve. The Youden index was employed to maximize both sensitivity and specificity. The optimal cut-off for sensitivity and specificity was chosen. The Kaplan-Meier survival analysis was utilized to investigate the impact of PNI categories on overall short- and long-term survival.

## Results

### Patient characteristics and clinical outcomes

The study enrolls 67 patients, with a mean age of 60.9  ±  12.4 years; 39 (58.3%) are male. The most common comorbid diseases were hypertension (65.4%), diabetes mellitus (26.9%), coronary artery disease (9%), and chronic obstructive pulmonary disease (COPD) (7.5%). The predominant etiologies of ESRD were hypertension (29.9%), idiopathic (25.4%), and diabetes mellitus (23.9%). Based on their PNI scores, four patients (6%) were classified as having severe malnutrition, 11 patients (16.4%) as having moderate malnutrition, and 52 patients (77.6%) as having normal nutritional status. The median dialysis duration as a month was 48 (24–84) months. During short and long-term follow-up, 7 (10.7%) of the cases died within a year, while 22 (32.8%) died within five years. Table 1 provides a summary of the patients’ other features.

**Table 1:** Patient characteristics and factors contributing to short- and long-term mortality.

	Overall Patients (n = 67)	Short term mortality (One-year mortality)			Long term mortality (Five-year mortality)		
		Dead (n = 7)	Alive (n = 60)	p-value	Dead (n = 22)	Alive (n = 45)	p-value
**Age, years**	60.9 ± 12.4	69.8 ± 6.2	59.9 ± 12.6	**0.046**	67.3 ± 9.1	57.8 ± 12.8	**0.003**
**Gender, male**	39 (58.3%)	5 (12.8%)	34 (87.2%)	0.454	17 (43.6%)	22 (56.4%)	**0.027**
**Smoking habit**							
* Ever-smoker*	31 (46.3%)	2 (6.5%)	29 (93.5%)	0.321	10 (32.3%)	21 (67.7%)	0.926
* Never-smoker*	36 (53.7%)	5 (13.9%)	31 (36.1%)		12 (33.3%)	24 (66.7%)	
**Cigarettes (pkg/year)**	20 (11–37)	40 (20–36)	20 (20–40)	0.387	30 (19–40)	20 (10–30)	0.105
**Hemodialysis duration, month**	48 (24–84)	36 (4–48)	48 (24–84)	0.207	36 (12–75)	48 (24–90)	0.403
**Comorbidity, n (%)**							
* Hypertension*	44 (65.7%)	5 (11.7%)	39 (88.6%)	0.737	18 (40.9%)	26 (59.1%)	**0.052**
* Diabetes Mellitus*	18 (26.9%)	1 (5.6%)	17 (94.4%)	0.428	9 (50%)	9 (50%)	0.070
* COPD*	5 (7.5%)	0	5 (100%)	0.427	1 (20%)	4 (66.7%)	0.525
* Coronary artery disease*	6 (9%)	0	6 (100%)	0.381	2 (33.3%)	4 (80%)	0.978
* Chronic liver failure*	2 (3%)	0	2 (100%)	0.624	1 (50%)	1 (50%)	0.600
**Etiologies of ESRD**							
* Hypertension*	20 (29.9%)	2 (10%)	18 (90%)	0.435	6 (30%)	14 (70%)	0.129
* Idiopathic*	17 (25.4%)	1 (5.9%)	16 (94.1%)		4 (23.5%)	13 (76.5%)	
* Diabetes mellitus*	16 (23.9%)	1 (6.3%)	15 (93.8%)		8 (50%)	8 (50%)	
* Polycystic*	11 (16.4%)	2 (18.2%)	9 (81.8%)		2 (18.2%)	9 (81.8%)	
* Glomerular diseases*	2 (3%)	1 (50%)	1 (50%)		2 (100%)	0	
* Nephrolithiasis*	1 (1.5%)	0	1 (100%)		0	1 (100%)	
**BMI, kg/m** ^2^	26.7 ± 5.1	27.3 ± 5.2	26.6 ± 5.1	0.722	27.1 ± 4.3	26.5 ± 5.4	0.657
**Spirometric Findings**							
* FEV1, L*	1.630 ± 0.660	0.987 ± 0.281	1.715 ± 0.653	**<0.001**	1.594 ± 0.607	1.661 ± 0.694	0.699
* FVC, L*	1.91 ± 0.77	1.044 ± 0.314	2.015 ± 0.745	**<0.001**	1.823 ± 0.733	1.957 ± 0.793	0.507
* FEV1/FVC*, %	87.2 ± 11.6	95.1 ± 2.7	90.0 ± 11.9	**<0.001**	89.6 ± 10.8	86.0 ± 12.1	0.238
**Body composition analysis**							
* Fat mass, kg*	20.7 (13.5–29.1)	24.1 (13.5–31.5)	20.1 (13.5–28.9)	0.532	21.5 (12.6–29.7)	20.7 (14.8–28.8)	0.889
* Muscle mass, kg*	44.1 ± 8.8	44.8 ± 7.2	43.9 ± 9.1	0.786	46.9 ± 7.5	42.6 ± 9.1	0.061
* Total body water, kg*	34.1 ± 5.5	33.9 ± 5.2	34.1 ± 5.5	0.915	35.8 ± 5.7	33.3 ± 5.2	0.083
* Fat-free mass, kg*	46.7 ± 7.5	46.5 ± 7.5	46.7 ± 7.6	0.927	49.1 ± 7.9	45.5 ± 7.1	0.068
* Fat-free mass index, kg/m* ^2^	7.9 (5.4–11.5)	10.1 (5.6–11.1)	7.6 (5.3–11.9)	0.992	49.5 (41.5–55.1)	43.8 (41.3–48.3)	0.123
**Initial laboratory findings**							
* CRP, mg/L*	6.2 (1.5–13.4)	16.3 (11.5–24.1)	4.9 (1.4–11.6)	**0.020**	10.1 (2.2–20.9)	4.2 (0.8–10.4)	**0.015**
* GFR*	7.50 ± 2.16	8.49 ± 1.32	7.39 ± 2.21	0.206	8.32 ± 1.57	7.10 ± 2.30	**0.028**
* Leukocyte count, per mm* ^3^	6985 ± 2180	7540 ± 2623	6920 ± 2139	0.481	6905 ± 2403	7024 ± 2089	0.836
* Lymphocyte count, per mm* ^3^	1458 ± 468	1374 ± 370	1467 ± 479	0.620	1264 ± 487	1552 ± 432	**0.017**
* Serum albumin, g/dl*	3.9 ± 0.3	3.6 ± 0.4	3.9 ± 0.3	0.080	3.7 ± 0.3	3.9 ± 0.3	**0.024**
* Serum transferrin, mg/dl*	1.6 ± 0.3	1.6 ± 0.3	1.6 ± 0.3	0.869	1.6 ± 0.2	1.6 ± 0.3	0.830
* proBNP, pg/ml*	427 (138–526)	955 (138–1007)	261 (138–456)	0.078	363 (131–930)	262 (168–425)	0.539
* Phosphorus, mg/dl*	5.1 ± 1.4	5.1 ± 1.8	5.1 ± 1.3	0.951	4.9 ± 1.2	5.1 ± 1.4	0.882
* TG, mmol/L*	189 (128–265)	190 (124–284)	184 (128–263)	0.838	144 (85–234)	207 (140–299)	**0.020**
* TCHO, mmol/L*	174.7 ± 39.3	174.7 ± 35.1	174.7 ± 40.1	0.998	176.4 ± 41.9	171.2 ± 34.1	**0.022**
* HDL, mmol/L*	32.5 ± 8.8	33.3 ± 5.8	33.9 ± 9.1	0.984	32.8 ± 9.9	34.1 ± 5.9	0.605
* LDL, mmol/L*	100.1 (81.9–124.5)	95.4 (76.1–132.4)	100.9 (83.7–123.2)	0.975	99.6 (80.4–121.9)	100.1 (82.7–125.1)	**0.031**
**PNI, total score**	39 (38–41)	37.7 ± 3.2	39.8 ± 3.5	0.083	38 (36–40)	39 (38–41)	0.002

Data are presented as mean ± SD, median (25–75), and n (%).

a. independent samples t-test, b. Mann-Whitney U test c. Pearson’s Chi-square test.

ESRD: End-stage renal disease, BMI: Body mass index, PNI: Prognostic Nutritional Index, FEV1: forced expiratory volume FVC: Forced vital capacity, FEV1/FVC: Forced expiratory volume ratio, CRP: C-reactive protein, ProBNP: pro b-type natriuretic peptide, GFR: glomerular filtration rate, TG: triglycerides, TCHO: total cholesterol, HDL: high-density lipoprotein, LDL: low-density lipoprotein.

The univariate analyses evaluating the factors on overall short-term mortality show a significant association with age, FEV1, FVC, FEV1/FVC, and CRP levels (respectively, p  =  0.046, p  <  0.001, p  <  0.001, p  <  0.001, p  =  0.020). Age, gender, CRP levels, GFR, lymphocyte count, serum albumin, triglycerides, total cholesterol, LDL, and PNI scores were significantly associated with long-term mortality (respectively, p  =  0.003, p  =  0.027, p  =  0.015, p  =  0.028, p  =  0.017, p  =  0.024, p  =  0.020, p =  0.022, p  =  0.031, p  =  0.002) (Table 1). Among patients, 5 (7.5%) were diagnosed with COPD. A comparative analysis of the PNI scores between patients with and without COPD revealed no statistically significant difference (38 [35–40] vs. 39 [38–41], p =  0.236).

### Diagnostic performance of PNI total scores for overall one-year and five-year mortality

**Fig 2:**
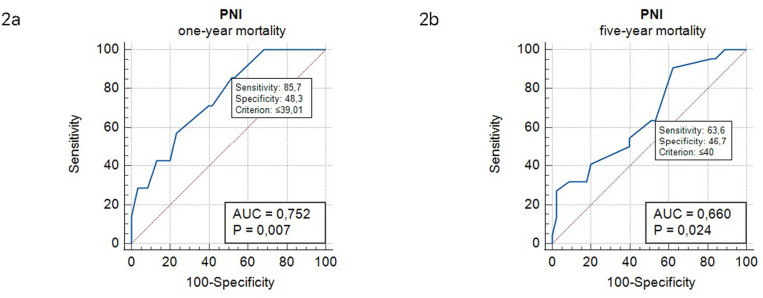
A and B. Diagnostic performance of PNI total score for overall one-year and five-year mortality.

The performance of the PNI total scores as a predictor of both short- and long-term mortality was assessed using ROC curve analysis (Fig 2a and 2b, and Table 2). The PNI total score’s optimal cut-off value for predicting short-term mortality was ≤  39.01, showing 85.7% sensitivity and 48.3% specificity, while for long-term mortality, the optimum cut-off value was ≤  40, with 90.9% sensitivity and 37.7% specificity (area under the curve =  0.750 and 0.660, respectively).

**Table 2:** Diagnostic performance of PNI total score for overall one-year and five-year mortality.

	Associated criterion	AUC	95% CI	Sensitivity (%)	95% CI	Specificity (%)	95% CI	p-value
**One-year mortality**								
*PNI, total score*	≤ 39.01	0.750	0.632–0.850	85.7	42.1–99.6	48.3	35.2–61.6	0.007
**Five-year mortality**								
*PNI, total score*	≤ 40	0.660	0.534–0.771	90.9	70.8–98.9	37.7	23,8–53,5	0.024

PNI: Prognostic Nutritional Index.

### Independent variables affecting one-year and five-year mortality

**Fig 3:**
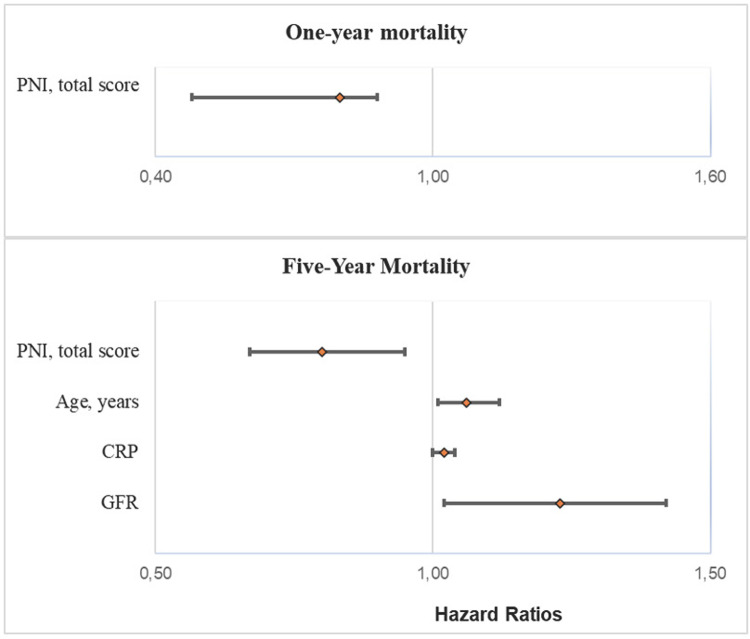
Independent variables affecting one-year and five-year overall mortality.

In the multivariate Cox regression analysis, the PNI total score (HR: 0.65; 95% CI: 0.48–0.88, p  =  0.006) predicted short-term mortality, while the PNI total score (HR: 0.80; 95% CI: 0.67–0.95, p  =  0.015), age (HR: 1.06; 95% CI: 1.01–1.12, p  =  0.021), and GFR (HR: 1.23; 95% CI: 1.02–1.42, p = 0.024) predicted long-term mortality (Fig 3 and Table 3).

**Table 3:** Independent variables affecting one-year and five-year overall mortality.

	HR	%95 CI	P value
**One-year mortality**			
* PNI, total score*	0.65	0.48–0.88	0.006
**Five-year mortality**			
* PNI, total score*	0.80	0.67–0.95	0.015
* Age, years*	1.06	1.01–1.12	0.021
* CRP*	1.02	1.00–1.04	0.054
* GFR*	1.23	1.02–1.42	0.024

CRP: C-reactive protein, PNI: Prognostic Nutritional Index, GFR: glomerular filtration rate.

### Kaplan-Meier survival curves for overall one-year and five-year mortality separated by nutritional categories

**Fig 4:**
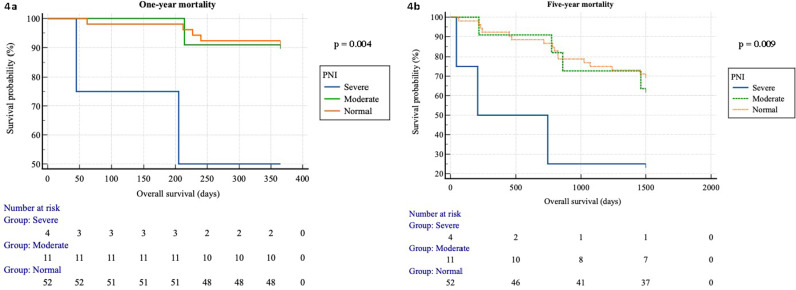
A and B. Kaplan-Meier survival curves for overall one-year and five-year mortality separated by PNI categories.

Survival times for PNI categories differed significantly depending on one-year and five-year mortality (respectively, p  =  0.004, p  = 0.01) (Fig 4a and 4b).

## Discussion

This study examined the prognostic significance of pulmonary dysfunction and the PNI score on short- and long-term mortality in ESRD patients undergoing maintenance hemodialysis. The results indicated that a higher PNI score significantly reduces the risk of both one-year and five-year mortality, while older age and higher GFR levels increase the risk of five-year mortality.

Protein-energy wasting (PEW) is prevalent among ESRD patients, particularly those undergoing dialysis [2]. PEW occurs in 28% to 54% of patients having maintenance dialysis [4]. The strong link between higher PNI scores and lower mortality is consistent with previous research emphasizing the importance of nutritional condition in ESRD patients [11]. The results of this study note that every increase in PNI score by one unit lowered the one-year mortality by 35% and five-year mortality by 20%. Short- and long-term follow-up revealed that 10.7% of cases died in less than a year, and 32.8% had died within five years. Optimal cut-off values were ≤  39.01 for the PNI total score in short-term mortality and ≤  40 for long-term mortality. High sensitivity and lower specificity characterize these cut-off values, especially in predicting long-term mortality.

The PNI score reflects the patient’s nutritional and immunological condition, including serum albumin and lymphocyte count [12]. Lymphocytopenia, an indicator of a weakened immune system, has important implications for the outcome of patients receiving hemodialysis [13]. Reddan et al. found that a low lymphocyte count was an independent predictor of mortality in hemodialysis patients [14]. According to earlier research, patients receiving hemodialysis who had high CRP levels also had higher fatality rates [15,16]. While the multivariate Cox model did not show any statistical significance, our analysis revealed that patients undergoing hemodialysis experienced poorer outcomes when their CRP levels were elevated.

The Prognostic Nutritional Index (PNI) serves as a prognostic biomarker for all-cause mortality in patients with type-2 diabetes [17], decompensated liver cirrhosis [18], and various cancers [19–21]. It also predicts the progression of diabetic nephropathy and is inversely related to the incidence of end-stage nephropathy [22]. Yu et al. observed that in patients with renal failure, a lower PNI is related to a higher risk of all-cause mortality [23]. Malnutrition reduces lymphocyte count by limiting the proteins required for their production and increasing susceptibility to infections, resulting in additional cell depletion [23].

Reduced lung function is common in this population due to fluid overload, uremic toxins, and coexisting conditions such as chronic COPD and heart failure [24,25]. Our findings indicate that impaired pulmonary function, as reflected by reduced FEV1 and FVC values, is significantly associated with short-term mortality in ESRD patients. This aligns with previous studies suggesting that lung dysfunction, influenced by fluid overload and uremic toxins, contributes to poor clinical outcomes in this population [8,9].

The study’s result that older age is associated with an increased risk of five-year mortality, with the probability of death rising by 6% with each year of age, is consistent with earlier research demonstrating that age is a crucial predictor of survival in ESRD patients. Predominant etiologies of ESRD in our study were hypertension (29.9%), idiopathic (25.4%), and diabetes mellitus (23.9%). As patients age, they accumulate an increasing number of comorbidities and experience a decrease in physiological reserve, which together contribute to elevated mortality rates. The finding underscores the necessity of age-specific management strategies in the treatment of elderly dialysis patients to reduce their elevated mortality risk.

Higher GFR is related to an increased risk of five-year mortality, with the risk of death increasing by 23% for every unit rise in GFR. Reduced GFR is typically indicative of renal dysfunction and is linked to an increased risk of death [26]. However, increased GFR may indicate hyperfiltration, which can occur in early diabetic nephropathy and other diseases marked by glomerular hypertension and increased filtration pressure [20]. Based on studies, renal hyperfiltration is associated with poor cardiovascular outcomes and an increased risk of death [27,28].

### Limitation

This study has several limitations that should be considered when interpreting the results. Firstly, the relatively small sample size may limit the results’ generalizability to the broader population of hemodialysis patients. The study did not evaluate the potential effects of medications like beta-blockers and diuretics, dietary intake or nutritional supplementation, or variations in dialysis parameters such as ultrafiltration rates and filter types, which could have influenced pulmonary function, nutritional status, and mortality outcomes. Furthermore, the retrospective design of this study precluded the real-time collection of data on dietary habits, pulmonary function, and mortality risk factors, which would have strengthened causal inferences. The lack of differentiation between cardiovascular, respiratory, and other causes of death complicates understanding how nutritional status and lung function specifically contribute to mortality.

Despite these limitations, the findings highlight the importance of nutritional and pulmonary assessments in predicting mortality among hemodialysis patients. Future research should aim to control for confounding factors and adopt more comprehensive clinical assessments to validate these results and enhance their applicability in clinical practice.

## Conclusion

Pulmonary function parameters and nutritional status emerge as critical predictors of short-term survival in hemodialysis patients, emphasizing the importance of integrated assessments in achieving optimal clinical outcomes. Furthermore, this study emphasizes the interplay of nutritional health, age, inflammation, and kidney function in determining both short- and long-term mortality risks. These findings emphasize the importance of a multidisciplinary approach to patient management, with comprehensive nutritional and pulmonary evaluations critical to improving survival and directing evidence-based therapeutic decision-making in this vulnerable population.
